# Minutes that matter: time-efficient high-intensity interval training improves cardiac function with transcriptomic evidence in post–myocardial infarction mice

**DOI:** 10.3389/fcell.2025.1728395

**Published:** 2026-01-12

**Authors:** Bing Bo, Chu Li, Aijing Guo, Ahmad Mujahid, Guandong Wang, Hui Zhang, Yanqing Shen, Wenli Cai

**Affiliations:** Department of Kinesiology, School of Physical Education and Sport, Henan University, Kaifeng, China

**Keywords:** bulk RNA-sequencing, cardiacfunction, cell-cycle-permissive state, exercise capacity, high-intensity interval training, myocardial infarction

## Abstract

High-intensity interval training (HIIT) improves cardiovascular performance, but the mechanisms remain incompletely delineated. We investigated whether HIIT improves left-ventricular (LV) remodeling after myocardial infarction (MI) in adult mice. Animals underwent permanent coronary ligation or sham surgery and were randomized to Control, HIIT-only, Sham, MI-only, and MI + HIIT. HIIT comprised 15 treadmill bouts (60 s at 90%–110% maximal running speed followed by 30 s rest), 3 days/week for 6 weeks. Baseline echocardiography 1 week after MI confirmed comparable LV dysfunction in MI-only and MI + HIIT groups. After intervention, the MI + HIIT group showed higher running capacity, improved LV ejection fraction (26.18% vs. 16.19%; p < 0.01) and fractional shortening (12.24% vs. 7.41%; p < 0.01), and less LV dilation versus MI-only. Myocardial fibrosis was reduced in MI + HIIT (8.85% vs. 13.17%; p < 0.01), consistent with physiological remodeling. 5-ethynyl-2′-deoxyuridine (EdU) incorporation identified more DNA synthesis in MI + HIIT (1.71%) and HIIT-only (1.24%) hearts. Bulk RNA sequencing showed coordinated upregulation of contractile and metabolic pathways and downregulation of apoptosis and inflammatory signaling, aligning with improved cell-cycle activity and oxidative–metabolic efficiency. Collectively, HIIT enhanced exercise capacity and cardiac function, attenuated fibrosis, and reprogrammed cardiac gene expression toward pro-contractile and anti-inflammatory programs consistent with a cell-cycle-permissive state in a post-MI mouse model.

## Graphical Abstract

**Figure:**
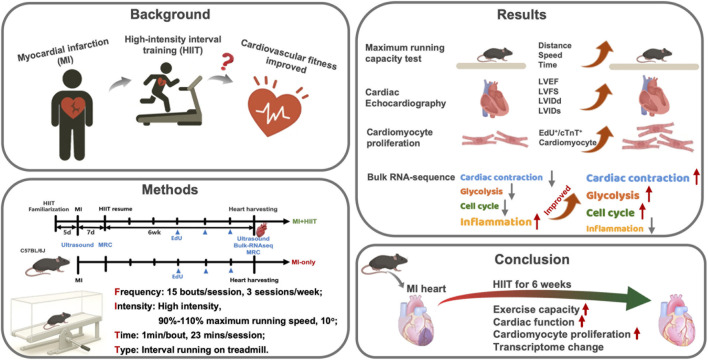

## 1 Introduction

Myocardial infarction (MI) remains a leading cause of morbidity and mortality worldwide. Recent estimates indicate that cardiovascular diseases account for approximately 19 million deaths annually, nearly one-third of all global deaths, with ischemic heart disease being the single largest contributor (Tsao et al., 2023; Martin et al., 2024). Survivors of MI frequently develop LV dysfunction and reduced exercise capacity, which is often assessed by maximal oxygen consumption (VO_2_max) or treadmill performance and is a strong independent predictor of cardiovascular and all-cause mortality in patients with coronary artery disease, including those after MI (Vanhees et al., 1994; Wisloff et al., 2005; Novakovic et al., 2022). After an acute MI, loss of cardiomyocytes in the infarct zone initiates a complex LV remodeling process characterized by dilation of the ventricle, compensatory hypertrophy of surviving myocytes, and progressive accumulation of interstitial and replacement fibrosis in both infarcted and remote myocardium. These structural changes are closely linked to impaired mitochondrial function, altered energetics, and increased oxidative stress, ultimately driving declines in systolic function and progression to heart failure (Frantz et al., 2022; Zhang et al., 2022). Improving cardiorespiratory fitness is therefore a central therapeutic goal, and structured exercise training is a key component of almost all contemporary cardiac rehabilitation programs (Dibben et al., 2023).

Beyond its systemic effects on hemodynamics and metabolism, exercise may also influence the intrinsic regenerative capacity of the heart. Preclinical studies suggest that endurance and interval exercise can induce cardiomyocyte DNA synthesis and cell-cycle activity in adult mammalian hearts (Vujic et al., 2018; Zhang et al., 2021; Lerchenmuller et al., 2022). Most of this evidence comes from healthy or pressure-overloaded hearts, however, and the extent to which similar exercise-induced cell-cycle responses can be elicited in the setting of MI remains much less clear (Cahill et al., 2017; Zhang et al., 2021). A better understanding of how exercise training modulates post-infarction LV remodeling, including cardiomyocyte cell-cycle status and the broader transcriptional landscape, could therefore inform future regenerative and rehabilitation strategies.

Currently, traditional moderate-intensity continuous training (MICT) remains the most widely prescribed mode of exercise for patients with cardiovascular disease, but many MICT regimens are based on general population guidelines rather than on evidence generated in patients with similar cardiac conditions (Li et al., 2021; Qin et al., 2022). In addition, patients with severe post-infarction LV remodeling and poor baseline fitness may find it difficult to sustain prolonged continuous exercise. HIIT, typically defined as an exercise modality that elicits ≥80% of VO_2_max in repeated high-intensity bouts interspersed with recovery periods, has emerged as a time-efficient alternative. In clinical and experimental settings, supervised HIIT typically elicits greater improvements in VO_2_max and submaximal exercise performance than work-matched MICT, particularly in patients with coronary artery disease or heart failure (Rognmo et al., 2004; Wisloff et al., 2007; Taylor et al., 2020; Qin et al., 2022; McGregor et al., 2023). Several trials and meta-analyses have also shown that HIIT can enhance vascular endothelial function, assessed by flow-mediated dilation, to a greater extent than MICT, and may promote more pronounced peripheral adaptations such as increased capillary density and mitochondrial content in skeletal and cardiac muscle (Wahl et al., 2022). These time-efficient physiological benefits provide a strong rationale for testing HIIT-based rehabilitation strategies after MI.

HIIT regimens are heterogeneous and can be grouped into at least eight categories. These classifications depend on the duration of the work interval (long ≥2 min vs. short <2 min), the ratio of work to rest intervals (fast ≥1 vs. slow <1), and the total session duration (high volume ≥15 min vs. low volume <15 min), which is determined by the number of work/rest pairs performed (Wahl et al., 2022). The benefits of HIIT are likely mediated by coordinated physiological changes in multiple organ systems. The optimal HIIT protocol for a given patient will depend on the disease being treated and the individual’s baseline fitness and tolerance. Long-interval HIIT protocols (e.g., 4-min bouts) have shown efficacy in clinically stable patients with coronary artery disease and post-infarction heart failure, as well as in rodent MI models (Rognmo et al., 2004; Wisloff et al., 2007; Wang et al., 2020). However, such regimens may be difficult to implement in patients with low cardiopulmonary reserve. Short-bout, low-volume HIIT protocols, composed of repeated very brief periods of high-intensity exercise separated by recovery intervals, may offer a more feasible and scalable option for individuals with limited exercise tolerance, but their effects on post-MI LV remodeling and underlying molecular mechanisms are not fully understood.

Therefore, we aimed to test whether a time-efficient HIIT program could benefit hearts with post-infarction LV remodeling. In this study, we used a mouse model of permanent coronary ligation to examine whether a short-bout, low-volume HIIT regimen could improve exercise capacity and LV function after MI. We also sought to investigate the mechanisms underlying HIIT by quantifying cardiomyocyte DNA synthesis as an index of cell-cycle activity and by performing bulk RNA sequencing to profile gene expression changes associated with reduced adverse remodeling, inflammation, and apoptosis in post-MI hearts, consistent with a more proliferation-permissive state. By integrating functional, structural, and transcriptomic data, our goal was to begin delineating how time-efficient HIIT might modulate adverse remodeling after MI and to provide mechanistic insight that could ultimately inform the design of HIIT-based rehabilitation strategies for post-MI patients.

## 2 Materials and methods

All data that support the findings of this work (Supplementary Tables S1–7), as well as a detailed account of the materials and methods used, are available in the Supplemental Materials. Additional technical information will be made available by the corresponding authors upon reasonable request. All animal experiments were conducted under the National Institutes of Health Guidelines for the Care and Use of Laboratory Animals (publication number 85-23, revised 1996) and approved by the Committee for the Ethics of Animal Experiments at Henan University (HUSOM2022-153). MI was induced by permanent ligation of the left anterior descending (LAD) coronary artery under isoflurane anesthesia (2% for induction, 1%–2% for maintenance in 100% oxygen), with body temperature maintained at 37 °C on a heated surgical platform, as described previously (Gao et al., 2010). The overall perioperative mortality associated with LAD ligation was 26.5%, and mortality did not differ appreciably between the MI-only and MI + HIIT groups (MI-only: 25.0%; MI + HIIT: 27.8%).

Eight-week-old male *C57BL/6J* mice were purchased from Charles River (Beijing, China) and maintained in a specific pathogen-free (SPF) laboratory animal facility at Henan University (Kaifeng, China). Only male mice were used in this study; female mice were not included, which is a common but important limitation in preclinical cardiovascular research. After baseline assessments, mice were randomly assigned to the five experimental groups (Control, HIIT-only, Sham, MI-only, and MI + HIIT) using a simple randomization procedure based on drawing lots, ensuring balanced group sizes. Animals in the MI + HIIT group underwent MI induction surgery and the HIIT regimen. Animals in the MI-only group underwent MI induction surgery without HIIT, HIIT-only animals underwent HIIT without previous MI induction, Sham mice underwent all surgical procedures for MI induction except coronary artery ligation and recovered without HIIT, and Control mice did not undergo any surgical procedures or HIIT.

Maximal running capacity (MRC) was assessed in each mouse using an incremental treadmill test on an 8-lane DB030 treadmill (Zhishuduobao Biological Company, Beijing) at 10° incline. Mice ran at 10 m/min for 10 min, after which the speed was increased by 2 m/min every 3 min until exhaustion, defined as the inability to remain on the treadmill belt for more than 5 s despite mild encouragement (Marcinko et al., 2015; Knudsen et al., 2020). The maximal speed reached in this test was taken as the maximal running speed (MRS), and the corresponding maximal running distance and time to exhaustion were used as indices of exercise capacity. The HIIT regimen was modified from a previously published protocol (Henriquez-Olguin et al., 2019; Knudsen et al., 2020) and consisted of 15 bouts per session, each comprising 60 s of running at 90%–110% of individual MRS followed by 30 s of passive recovery. Sessions were performed on three non-consecutive days per week for 6 weeks, and MRS was increased by 1 m/min each week to maintain high exercise intensity throughout the training period.

Cardiac function and remodeling were assessed echocardiographically by an investigator blinded to group allocation. Transthoracic echocardiography was performed using a Vevo 2100 imaging system (VisualSonics, Toronto, Canada) equipped with an MS-550D 40-MHz linear-array transducer under light isoflurane anesthesia (1%–1.5%), with heart rate maintained between 450 and 550 beats/min. Infarct size was measured via Masson’s trichrome staining, and cardiomyocyte DNA synthesis and cell-cycle activity were quantified by analysis of 5-ethynyl-2′-deoxyuridine (EdU) incorporation (Patterson et al., 2017) by observers blinded to group identity. Differential gene and pathway activity was evaluated via RNA sequencing (RNA-seq) of LV tissue followed by downstream bioinformatic analyses. Raw reads were quality-checked, trimmed, aligned to the mouse reference genome, and quantified at the gene level; differential expression was assessed using DESeq2, and significantly differentially expressed genes (DEGs; false discovery rate [FDR] < 0.05) were subjected to Gene Ontology (GO) and Kyoto Encyclopedia of Genes and Genomes (KEGG) pathway enrichment analysis using the clusterProfiler package in R. Enrichment results were visualized with ggplot2 and heatmap-based plots, and pathways with Benjamini–Hochberg–adjusted p-values (FDR) < 0.05 were considered significantly enriched.

Statistical analyses were performed with MATLAB software (version 2024a, MathWorks, Natick, MA), and results are presented as mean ± standard deviation (SD) unless otherwise specified. Normality and homogeneity of variance were evaluated with the Shapiro–Wilk test and Levene’s test, respectively. For single time-point comparisons across groups (e.g., Week 6 histological and molecular endpoints), group differences were assessed using one-way ANOVA. When a significant main effect of group was detected, *post hoc* pairwise comparisons between groups were performed using independent two-tailed t-tests, and the resulting p-values were adjusted for multiple testing using the Benjamini–Hochberg false discovery rate (FDR) procedure. For variables measured at two time points in the same animals (exercise capacity and echocardiographic parameters at Week 0 and Week 6), two-way repeated-measures ANOVA was additionally performed with group (Control, HIIT-only, Sham, MI-only, and MI + HIIT) as the between-subject factor, time (Week 0 vs. Week 6) as the within-subject factor, and a group × time interaction term. When indicated, *post hoc* pairwise comparisons at specific time points were conducted with FDR-adjusted p-values as described above. A p-value or FDR-adjusted p-value <0.05 was considered statistically significant.

## 3 Results

### 3.1 HIIT improved exercise capacity and cardiac performance in mice with infarcted hearts

**FIGURE 1:**
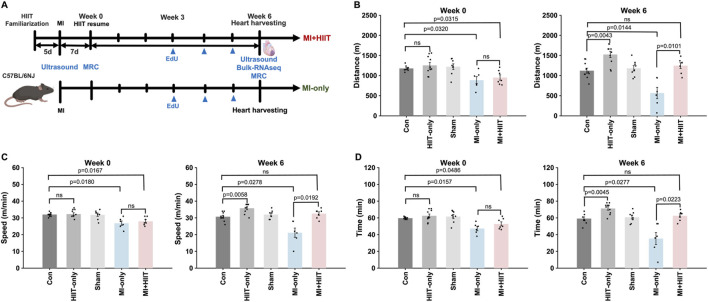
HIIT improved exercise capacity in mice with infarcted hearts. **(A)** The experimental protocol is illustrated as a schematic. Mice were familiarized with the treadmill apparatus for 5 days; then, mice in the Sham, MI-only, and MI + HIIT groups underwent the corresponding surgical procedures, and all animals remained sedentary for 1 week. The HIIT regimen was initiated in HIIT-only and MI + HIIT mice at Week 0 and continued until Week 6, while animals in the other three groups remained sedentary. **(B–D)** Exercise capacity was assessed by measuring running **(B)** distance, **(C)** speed, and **(D)** time to exhaustion at Week 0 and Week 6. Data are presented as mean ± SD. Group differences were evaluated using one-way ANOVA for comparisons across all five experimental groups, followed by *post hoc* analysis where applicable. For comparisons between two groups, independent two-tailed t-tests were performed, with p-values adjusted for multiple comparisons using the Benjamini-Hochberg false discovery rate (FDR) correction. An adjusted p-value of less than 0.05 was considered statistically significant. The sample size for each group was: Control (n = 10), HIIT-only (n = 10), Sham (n = 8), MI-only (n = 7), MI + HIIT (n = 7). EdU, 5-ethynyl-2′-deoxyuridine; HIIT, high-intensity interval training; MI, myocardial infarction; MRC, maximum running capacity.

Mice were familiarized with the treadmill apparatus by running them 5 min per day (5 m/min, 0° incline) for 5 days before surgical procedures were performed; then, the exercise routine was paused for 1 week in all groups as the Sham, MI-only, and MI + HIIT group recovered from surgery. Animals in the HIIT-only and MI + HIIT groups began the HIIT regimen at Week 0 and continued until Week 6 while animals in the other three groups (Control, Sham, and MI-only) remained sedentary (Figure 1A). Week 0 measurements of running distance (Figure 1B), speed (Figure 1C), and time to exhaustion (Figure 1D) were significantly lower in the MI-only group than in Control group but did not differ significantly between the MI-only and MI + HIIT group or among the three groups that did not undergo MI induction surgery. At Week 6, however, all three parameters were significantly greater in the HIIT-only group than in Control group, while measurements in the MI + HIIT group were significantly greater than in MI-only mice and were similar to Control or Sham groups. Measurements also increased from Week 0 to Week 6 in the HIIT-only and MI + HIIT groups, while measurements in the MI-only group declined (Supplementary Tables S1,2).

**FIGURE 2:**
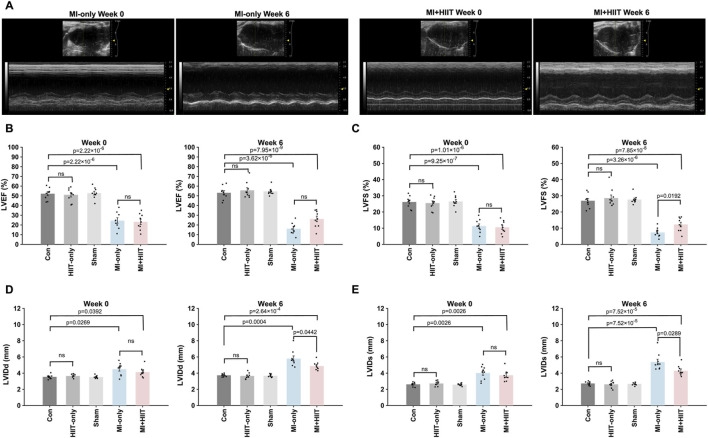
HIIT improved heart function and limited adverse cardiac remodeling in mice with infarcted hearts. **(A)** Echocardiographic images were collected at Week 0 and Week 6 and used to determine measurements of left ventricular **(B)** ejection fraction (LVEF) and **(C)** fractional shortening (LVFS), left-ventricular internal dimensions at **(D)** diastole (LVIDd) and **(E)** systole (LVIDs). Representative echocardiographic images are displayed in A for mice in the MI-only and MI + HIIT groups at both time points. Data are presented as mean ± SD. Group differences were evaluated using one-way ANOVA for comparisons across all five experimental groups, followed by *post hoc* analysis where applicable. For comparisons between two groups, independent two-tailed t-tests were performed, with p-values adjusted for multiple comparisons using the Benjamini-Hochberg false discovery rate (FDR) correction. An adjusted p-value of less than 0.05 was considered statistically significant. (n = 9 mice per experimental group).

The results from our exercise capacity studies were generally consistent with those from echocardiographic assessments (Figure 2A; Supplementary Figure S1; Supplementary Tables S3, 4) of cardiac function. At Week 0, measurements of left-ventricular (LV) ejection fraction (LVEF, Figure 2B) and fractional shortening (LVFS, Figure 2C) were significantly smaller, while LV internal dimensions at diastole and systole (LVIDd, Figure 2D, and LVIDs; Figure 2E, respectively) were significantly greater, in MI-only animals than in the Control group, but there were no significant differences between MI-only and MI + HIIT group or among the three groups that did not undergo MI induction in any parameter. In MI-only group, echocardiographic measurements continued to worsen between Week 0 and Week 6, with further reductions in LVEF and LVFS and progressive increases in LVIDd and LVIDs. By contrast, these parameters improved over the same period in MI + HIIT group, such that at Week 6 all four indices were significantly better in the MI + HIIT group than in the MI-only group.

To account for the repeated measurements at Week 0 and Week 6, we additionally analyzed exercise capacity and echocardiographic parameters using two-way repeated-measures ANOVA with group as the between-subject factor and time as the within-subject factor. For exercise capacity, these analyses revealed significant group × time interactions for maximal running speed (F (4,36) = 8.05, p = 0.0001), running distance (F (4,36) = 7.69, p = 0.0001), and time to exhaustion (F (4,36) = 6.42, p = 0.0005), indicating that performance deteriorated over time in the MI-only group but improved markedly in the MI + HIIT and HIIT-only groups, with minimal changes in the Control and Sham groups. For echocardiographic variables, a significant group × time interaction was observed for LVEF (F (4,40) = 3.20, p = 0.023), and a similar pattern was seen for LVFS (F (4,40) = 2.42, p = 0.064), with declines in the MI-only group and relative preservation or slight improvement in the MI + HIIT group. Group × time interactions were highly significant for LV dimensions, both LVIDd (F (4,40) = 6.57, p = 0.0004) and LVIDs (F (4,40) = 6.76, p = 0.0003), reflecting progressive dilatation in the MI-only group and attenuated increases in chamber size in the MI + HIIT group, with minimal changes in non-infarcted groups. These repeated-measures analyses support the conclusion that HIIT not only improved endpoint measurements of exercise capacity and LV function but also altered their trajectories over the 6-week intervention period.

### 3.2 HIIT increased cardiac mass, reduced infarct size, and promoted DNA synthesis in mice with infarcted hearts

**FIGURE 3:**
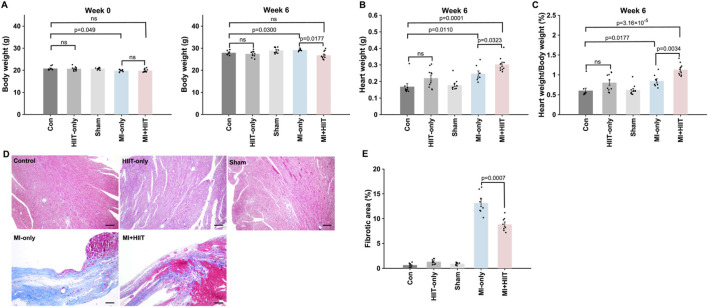
HIIT increased myocardial mass and reduced fibrosis in infarcted mouse hearts. **(A)** Mouse bodyweights were measured at Week 0 and immediately after animals were sacrificed at Week 6. **(B)** Heart weights were measured after harvesting at Week 6, and **(C)** heart-weight-to-bodyweight ratios were calculated and expressed as a percentage. **(D)** Cardiac tissue sections were obtained from the LVs of mice sacrificed at Week 6 and stained with Masson’s trichrome to identify fibrotic (blue) and nonfibrotic (red) regions; then, **(E)** the ratio of the area of fibrosis to the area of the entire LV was calculated and presented as a percentage. Representative images are displayed in panel D (Scale bar = 100 μm). Data are presented as mean ± SD. Group differences were evaluated using one-way ANOVA for comparisons across all five experimental groups, followed by *post hoc* analysis where applicable. For comparisons between two groups, independent two-tailed t-tests were performed, with p-values adjusted for multiple comparisons using the Benjamini-Hochberg false discovery rate (FDR) correction. An adjusted p-value of less than 0.05 was considered statistically significant. (n = 8–10 mice per experimental group).

Body weights for all five experimental groups were similar at Week 0, but MI + HIIT group was significantly lighter than MI-only group at Week 6 (Figure 3A). Heart weights (HW, Figure 3B) and heart-weight-to-bodyweight ratios (HW:BW, Figure 3C) were significantly greater in MI + HIIT group than in MI-only group at Week 6 (Supplementary Tables S5), which suggests that the HIIT regimen increased cardiac hypertrophy in the MI + HIIT group; however, HW and HW:BW ratios at Week 6 also tended to be greater (but not significantly) in HIIT-only group than in Control or Sham groups. To verify that the extent of infarction was comparable between groups at the start of the training protocol, a separate cohort of mice was sacrificed at Week 0 for histological assessment. Masson’s trichrome staining showed similar infarct size, expressed as percentage of LV area, in the MI-only and MI + HIIT groups (9.74% ± 1.68% vs. 9.97% ± 1.47%, p = 0.77; Supplementary Figure S2; Supplementary Tables S6), indicating comparable baseline infarct burden in these two MI groups. At Week 6, LV sections collected (Figure 3D) from the MI-only group were significantly more fibrotic than those from the Control, HIIT-only, and Sham groups, measurements of LV fibrosis in the MI + HIIT group were significantly lower than in the MI-only groups and did not differ significantly from measurements in the three groups that did not undergo MI induction (Figure 3E).

**FIGURE 4:**
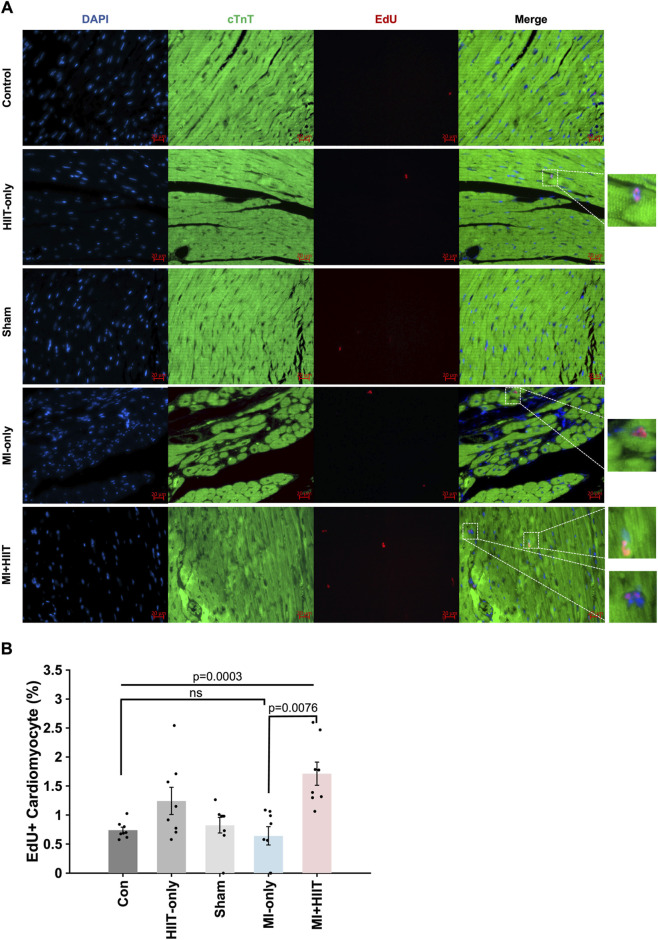
HIIT increased cardiomyocyte DNA synthesis and cell-cycle activity in infarcted mouse hearts. Mice in all groups received intraperitoneal injections of 5-ethynyl-2′-deoxyuridine (EdU) once per week at Weeks 3, 4, and 5; then, after sacrifice at Week 6, **(A)** sections of LV tissue were stained for the presence of EdU (red), for the expression of cardiac troponin T (cTnT, green) to visualize cardiomyocytes, and with Hoechst 33,342 (blue) to label nuclei. **(B)** Cardiomyocyte proliferation was summarized as the proportion of cTnT-expressing cells that were also positive for EdU and expressed as a percentage. Representative images are displayed in panel A (Scale bar = 20 μm). Data are presented as mean ± SD. Group differences were evaluated using one-way ANOVA for comparisons across all five experimental groups, followed by *post hoc* analysis where applicable. For comparisons between two groups, independent two-tailed t-tests were performed, with p-values adjusted for multiple comparisons using the Benjamini-Hochberg false discovery rate (FDR) correction. An adjusted p-value of less than 0.05 was considered statistically significant. (n = 8 mice per experimental group).

Furthermore, the proportion of cardiomyocytes that were positive for EdU incorporation (Figure 4A) was significantly greater in the MI + HIIT group than in the MI-only group, and in the HIIT-only group than in the Control or Sham groups (Figure 4B). Collectively, these observations suggest that the HIIT regimen increased myocardial mass and limited fibrosis in infarcted mouse hearts, and that the hypertrophy observed in MI + HIIT group was, at least in part, a physiological adaptation to exercise; in addition, the higher fraction of EdU-labeled cardiomyocyte nuclei reflects enhanced DNA synthesis and cell-cycle activity (S-phase entry).

### 3.3 HIIT altered the activity of pathways that regulate contractile activity, metabolism, cell cycle activity, apoptosis, and inflammation in infarcted mouse hearts

**FIGURE 5:**
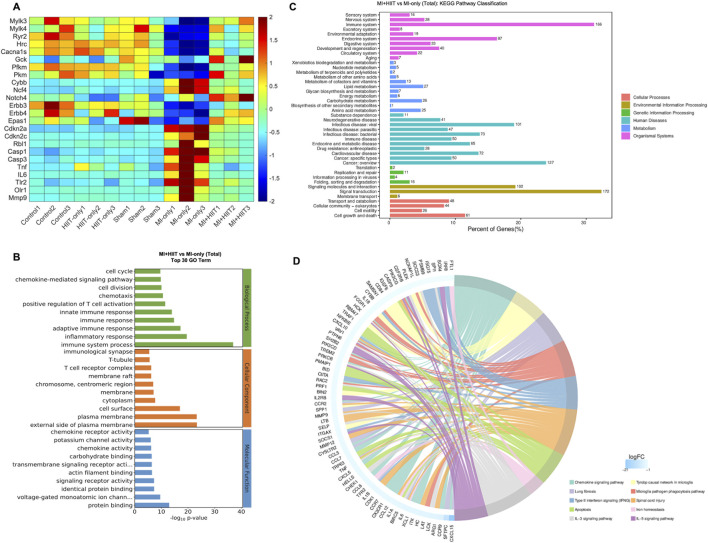
Gene Ontology enrichment and KEGG Pathway analysis of bulk RNA sequencing data suggest that HIIT altered the activity of pathways that regulate myocardial contractility, cardiac metabolism, cardiomyocyte proliferation, apoptosis, and inflammation in infarcted mouse hearts. Bulk RNA sequencing analysis was performed with LV tissues collected after sacrifice at Week 6 (n = 3 mice per experimental group). **(A)** The abundance of expression for genes that improve cardiac contraction (Mylk3, Mylk4, Ryr2, Hrc, Cacna1s), increase myocardial metabolic efficiency (Gck, Pfkm, Pkm, Cybb, Ncf4), promote (Notch4, Erbb3, Erbb4, Epas1) or inhibit (Cdkn2a, Cdkn2c, Rbl1) cardiomyocyte proliferation, and reduce apoptosis or promote inflammation (Casp1, Casp3, Tnfα, Il6, Tlr2, Olr1, Mmp9) is summarized as a heat map. **(B)** Data for the MI-only and MI + HIIT groups were compared via Gene Ontology (GO) enrichment analysis, and the 30 terms that were most significantly (p < 0.05) upregulated in the MI + HIIT group were identified. **(C)** Data for the MI-only and MI + HIIT groups were compared via KEGG pathway analysis to identify pathway classifications that were significantly (p < 0.05) upregulated in the MI + HIIT group; for each pathway, the x-axis represented the percentage of pathway genes that were differentially expressed between the MI-only and MI + HIIT groups. **(D)** Genes that were specifically associated with the indicated cellular and physiological processes are displayed as a KEGG chord diagram. All p-values were adjusted by the Benjamini-Hochberg false discovery rate (FDR) correction.

Bulk RNA-seq analysis of myocardial tissue (Supplementary Figure S3; Supplementary Tables S7, 8) indicated that HIIT after MI induced widespread changes in gene expression (Figure 5A). First, genes linked to cardiac contractile activity and calcium handling (e.g., Mylk3, Mylk4, Ryr2, Hrc, and Cacna1s) were upregulated, consistent with enhanced myocardial contraction. Second, metabolic genes such as Gck, Pfkm, and Pkm were upregulated, whereas Cybb and Ncf4 were downregulated, suggesting improved metabolic efficiency and reduced oxidative stress. Third, we observed upregulation of cell-cycle–associated genes (e.g., Notch4, Erbb3, Erbb4, and Epas1) together with downregulation of cell-cycle inhibitors (e.g., Cdkn2a, Cdkn2c, and Rbl1), consistent with a more cell-cycle–permissive myocardial state. Finally, pro-apoptotic and pro-inflammatory genes (e.g., Casp1, Casp3, Tnfα, Il6, Tlr2, Olr1, and Mmp9) were downregulated, indicating attenuated apoptotic and inflammatory signaling in MI + HIIT hearts.

When RNA-seq data from MI + HIIT and MI-only groups were evaluated via GO analysis, genes associated with cell-cycle regulation, myocardial contraction, metabolic processes, and immune responses were significantly enriched in the MI + HIIT group (Figure 5B). KEGG pathway analysis further showed enrichment of pathways related to signal transduction, energy metabolism, and cardiovascular function (Figure 5C), and highlighted numerous genes involved in cell-cycle regulation, myocardial contraction, and inflammation that were upregulated in MI + HIIT hearts (Figure 5D).

**FIGURE 6:**
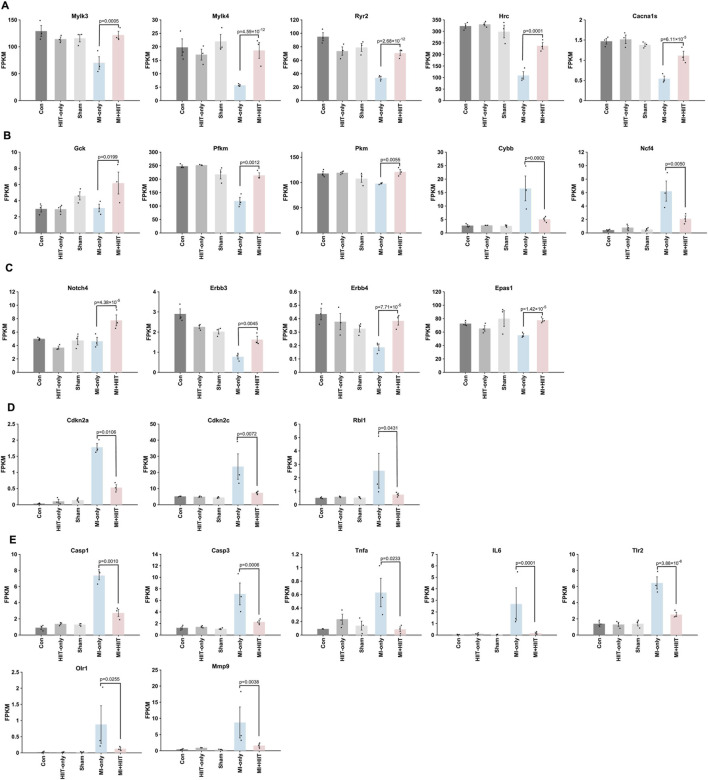
FPKM normalization of bulk RNA sequencing data suggests that HIIT led to changes in gene expression that are associated with increases in cardiomyocyte proliferation, improvements in myocardial contractility and metabolism, and declines in apoptosis and inflammation in infarcted mouse hearts. Bulk RNA sequencing data were normalized according to Fragments Per Kilobase of transcript per Million mapped reads (FPKM), and results are displayed for **(A)** genes that contribute to cardiac contractile activity (Mylk3, Mylk4, Ryr2, Hrc, Cacna1s), **(B)** genes that regulate myocardial metabolism (Gck, Pfkm, Pkm, Cybb, Ncf4), **(C)** genes that promote cardiomyocyte proliferation (Notch4, Erbb3, Erbb4, Epas1), **(D)** genes that inhibit cardiomyocyte cell-cycle activity (Cdkn2a, Cdkn2c, Rbl1), and **(E)** genes that promote inflammation or apoptosis (Casp1, Casp3, Tnfα, Il6, Tlr2, Olr1, Mmp9). Data are presented as mean ± SD; p-values were determined via independent two-tailed t-tests, with p-values adjusted for multiple comparisons using the Benjamini-Hochberg false discovery rate (FDR) correction. An adjusted p-value of less than 0.05 was considered statistically significant. (n = 3 mice per experimental group).

Notably, similar patterns were observed when expression data were normalized as fragments per kilobase of transcript per million mapped reads (FPKM). Compared with hearts from MI-only animals, hearts from the MI + HIIT group expressed higher levels of genes involved in myocardial contraction (Mylk3, Mylk4, Ryr2, Hrc, and Cacna1s; Figure 6A) and higher levels of cell-cycle–related genes associated with a proliferation-permissive phenotype (Notch4, Erbb3, Erbb4, and Epas1; Figure 6C), together with lower levels of the cell-cycle inhibitors Cdkn2a, Cdkn2c, and Rbl1 (Figure 6D). The HIIT regimen also induced changes in genes linked to cardiac metabolism and mitochondrial activity, with upregulation of Gck, Pkm, and Pfkm and downregulation of Cybb and Ncf4 (Figure 6B). In addition, apoptosis- and inflammation-related genes (Casp1, Casp3, Tnfα, Il6, Tlr2, Olr1, and Mmp9) were consistently downregulated in MI + HIIT hearts (Figure 6E). Together, these FPKM-normalized analyses support the conclusion that HIIT shifted the transcriptomic landscape towards enhanced contractile and metabolic capacity with reduced inflammatory and apoptotic signaling after MI.

## 4 Discussion

Exercise is considered a key component of cardiac rehabilitation for patients with myocardial disease. A recent multicentre, randomized clinical trial showed that HIIT (n = 187) was safe and more effective than MICT (n = 195) for improving cardiorespiratory fitness in patients with clinically stable coronary artery disease (CAD) (McGregor et al., 2023). In clinical populations, HIIT is generally defined as an exercise regimen that elicits ≥80% VO_2_max, but the diversity of prescribed exercise protocols can lead to a wide range of patient outcomes (Li et al., 2021). Regimens with long work intervals (e.g., 4 min) have been effective in studies of clinically stable patients with CAD (Rognmo et al., 2004) or post-infarction heart failure (Wisloff et al., 2007), as well as a rat MI model (Wang et al., 2020). However, such protocols are unlikely to be suitable for patients with low cardiopulmonary fitness (Li et al., 2021). Here, we show that a short-duration protocol consisting of 15 daily 1.5-min bouts (60 s running followed by 30 s of rest) can effectively increase exercise capacity and improve cardiac performance in a mouse MI model. Compared with a previous rodent MI study (Wang et al., 2020), our regimen used a shorter intervention period and reduced weekly frequency (3 days/week for 6 weeks vs. 5 days/week for 8 weeks), which may better reflect what is feasible for many post-MI individuals. We observed significant improvements in all key measures of exercise capacity, including running distance, speed, and time to exhaustion. These changes suggest that HIIT increased not only muscular strength but also endurance and resistance to fatigue. Notably, impairments in exercise capacity are among the most debilitating symptoms in patients with MI (Tashiro et al., 2020). Improvements in these parameters are associated with reductions in adverse cardiac events and hospitalization rates (Novakovic et al., 2022), underscoring the potential clinical relevance of the functional benefits observed in our MI + HIIT mice.

Cardiopulmonary adaptations are a primary target when prescribing HIIT, but they are not the only clinically relevant goal, particularly for patients with CAD and MI. Adverse ventricular remodeling and impaired systolic function remain persistent therapeutic challenges in this population (Frantz et al., 2022; Yap et al., 2023). In our study, echocardiographic assessments indicated that the short-duration HIIT regimen improved indices of LV function (LVEF and LVFS) and attenuated chamber dilatation, as reflected by smaller LVIDd and LVIDs in MI + HIIT mice compared with MI-only mice. This anti-dilatory effect may be particularly important because ventricular dilatation is strongly correlated with increased wall stress, reduced contractile performance, and progression to heart failure (Lazzeroni et al., 2016; Camici et al., 2020). Furthermore, HW:BW ratios were higher in MI + HIIT mice than in the MI-only group, and a similar, albeit non-significant, trend was observed in HIIT-only versus Control animals. When considered alongside the functional benefits associated with HIIT, this pattern is consistent with predominantly adaptive rather than maladaptive ventricular growth. Supporting this interpretation, our RNA-seq analysis revealed that the hypertrophy-inhibitory marker Rcan1 was significantly upregulated following HIIT in MI hearts, whereas classical pathological hypertrophy markers (Nppa, Nppb, and Nppc) tended to be lower, although these differences did not reach statistical significance (Supplementary Figure S4). Together, these findings suggest that the observed increase in cardiac mass in MI + HIIT mice may, at least in part, reflect physiological remodeling rather than overt pathological hypertrophy.

The limited capacity of adult mammalian cardiomyocytes to re-enter the cell cycle is a key barrier to myocardial repair and contributes to adverse remodeling after MI (Cahill et al., 2017). In our study, EdU incorporation was significantly higher in cardiomyocytes from MI + HIIT group than in those from MI-only group, indicating enhanced DNA synthesis and cell-cycle activity (S-phase entry) (Vujic et al., 2018). EdU-labelling was also greater in HIIT-only hearts than in Control or Sham groups. Consistently, bulk RNA-seq revealed transcriptional changes in MI + HIIT hearts enriched for cell-cycle regulation pathways together with gene programs previously associated with cardiomyocyte cell-cycle engagement (Collesi et al., 2008; Ebelt et al., 2008; Bersell et al., 2009; Hao et al., 2014). These findings suggest that HIIT may mitigate post-infarction LV remodeling, in part, by promoting cardiomyocyte cell-cycle activity, while not providing direct evidence of mitosis or cytokinesis. Notably, HIIT after MI also triggered transcriptomic changes associated with improvements in contractile performance (Uurasmaa et al., 2021) and metabolic efficiency (Bei et al., 2024), as well as declines in inflammation and apoptosis, which likely contributed to attenuation of adverse remodeling and facilitation of cardiac repair (Frangogiannis, 2012; Zhang et al., 2022; Hilgendorf et al., 2024; MoTr et al., 2024).

Taken together, these findings provide a mechanistic framework for thinking about how HIIT might be leveraged in human post-MI rehabilitation. At the organ level, the combination of improved exercise capacity, enhanced LV systolic function, and attenuated dilation and fibrosis suggests that time-efficient HIIT can shift the balance of remodeling towards a more favorable phenotype. At the cellular and molecular level, the transcriptomic signature identified in MI + HIIT hearts—characterized by upregulation of genes involved in contractile machinery, calcium handling, and mitochondrial metabolism and downregulation of inflammatory and apoptotic pathways—points to specific biological processes that may be engaged by appropriately prescribed high-intensity intervals. Although direct extrapolation from mice to patients is not possible, these data support the concept that short-bout, low-volume HIIT protocols could be particularly valuable for post-MI patients with limited exercise tolerance, and they highlight candidate pathways for future biomarker, imaging, and interventional studies in human cardiac rehabilitation.

## 5 Study limitations

The limitations of this investigation include the use of a murine MI model, which cannot fully replicate human physiology or the complexity of clinical cardiac rehabilitation programs. Accordingly, our findings should be regarded as mechanistic and hypothesis-generating rather than directly prescriptive for patient care. In particular, whether similar improvements in LV contractile function and remodeling, reductions in inflammatory and apoptotic signaling, and changes in cardiomyocyte cell-cycle activity can be elicited by short-bout, low-volume HIIT in post-MI patients, and how these adaptations might best be targeted in clinical exercise prescriptions, will need to be addressed in future clinical trials. Furthermore, only male mice were studied; potential sex-specific differences in the response to HIIT after MI were not addressed and warrant further investigation. All animals in the HIIT-only and MI + HIIT groups underwent the same HIIT regimen, and the duration of the study was limited to 6 weeks, so additional investigations are necessary to determine whether the regimen requires further optimization and whether the observed benefits persist over longer time periods. In addition, blinding was not feasible during supervision of the HIIT sessions themselves, which may introduce some potential performance bias, although objective quantitative endpoints and predefined analysis protocols were used to minimize this. Moreover, EdU labelling identifies S-phase entry and, by itself, does not confirm mitosis or cytokinesis; corroboration with mitotic/cytokinetic markers and, ideally, lineage-tracing strategies is required to establish *bona fide* cardiomyocyte division. Finally, our bulk RNA-seq analysis was performed in a relatively small number of animals (n = 3 per group) and should be considered exploratory and hypothesis-generating rather than definitive; the observed transcriptomic changes will need to be validated and functionally tested in targeted gain- and/or loss-of-function studies both *in vitro* and *in vivo*.

## 6 Conclusion

In conclusion, a HIIT regimen of 15 bouts of 60-s running interleaved with 30-s rest, performed 3 days per week for 6 weeks, significantly improved exercise capacity and cardiac function in a murine MI model. These functional gains coincided with attenuation of adverse remodeling (reduced dilation and fibrosis) and with increased cardiomyocyte cell-cycle activity. Parallel transcriptomic shifts favored enhanced contractile machinery and metabolic efficiency with downregulated apoptotic and inflammatory signaling. Collectively, these preclinical findings support the concept that time-efficient HIIT may represent a promising rehabilitation strategy for ischemic heart disease, particularly for individuals with limited tolerance for prolonged endurance exercise. However, they should be regarded as exploratory and hypothesis-generating, and confirmatory studies using mitotic/cytokinetic markers, longer-term follow-up, and carefully designed clinical trials will be required to determine how best to translate these observations into exercise prescriptions for post-MI patients.

## Data Availability

The datasets presented in this study can be found in online repositories. The names of the repository/repositories and accession number(s) can be found in the article/Supplementary Material.
