# Supplementary material for: Minutes that matter: time-efficient high-intensity interval training improves cardiac function with transcriptomic evidence in post–myocardial infarction mice
Source: Front Cell Dev Biol. 2026 Jan 12;13:1728395. doi: 10.3389/fcell.2025.1728395 (PMC12832661; doi:10.3389/fcell.2025.1728395)
Supplement: Supplementary file 1 [file DataSheet1.pdf]

Supplementary Material

Minutes that matter: Time-efficient High-intensity interval training improves post–myocardial infarction cardiac function with transcriptomic evidence in mice

Bing Bo[1], Chu Li[1], Aijing Guo[1], Ahmad Mujahid[1], Guandong Wang[1], Hui Zhang[1], Yanqing Shen[1], and Wenli Cai[1*]

[1] Department of Kinesiology, School of Physical Education and Sport, Henan University, Kaifeng, China

*** Correspondence:**
Wenli Cai
10180085@vip.henu.edu.cn
Yanqing Shen
shenyanqing@henu.edu.cn
Hui Zhang
10180048@vip.henu.edu.cn

1 Supplemental Methods

1.1 Experimental groups

Eight-week-old, male, C57BL/6J mice were purchased from Charles River (Beijing, China), and randomly distributed into five experimental groups. Each group consists of n>7 animals (Supplemental Tables 1, 4). Animals in the MI+HIIT group underwent surgically induced myocardial infarction (MI) and the high-intensity interval training (HIIT) regimen, animals in the MI-only group underwent MI induction surgery without HIIT, HIIT-only animals underwent HIIT without previous MI induction, Sham mice underwent all surgical procedures for MI induction except coronary artery ligation and recovered without HIIT, and Control mice did not undergo any surgical procedures or HIIT.

1.2 Mouse MI model

Mice were anesthetized using a precision vaporizer to deliver 2% isoflurane in 100% oxygen for induction and 1–2% for maintenance; adequate anaesthesia was confirmed by the absence of a withdrawal reflex to paw pinch. Mice were placed in a supine position on a heated surgical platform to maintain a body temperature of 37°C; then, the chest area was shaved, and the skin was disinfected with 70% ethanol and povidone-iodine solution. A left thoracotomy was performed at the fourth intercostal space to expose the heart, and the ribs were separated with retractors to provide a clear view of the left ventricle (LV). The left anterior descending (LAD) coronary artery was visually identified under a surgical microscope, and a 6-0 polypropylene suture was passed under the LAD artery 1–2 mm below the left atrial appendage and tied securely to induce permanent occlusion. Occlusion was

confirmed by blanching of the LV wall distal to the ligation site; then, the thoracic cavity was closed in layers with 5-0 absorbable sutures used for muscle tissue and 6-0 nonabsorbable sutures used for the skin. Mice recovered on a heating pad under close observation until fully awake. Following LAD ligation surgery, mice received post-operative analgesia and antibiotics to minimize pain and prevent infection. Buprenorphine (0.1 mg/kg subcutaneously) was administered every 12 hours for 72 hours to ensure adequate analgesia. To prevent infection, enrofloxacin (10 mg/kg, subcutaneous) was provided for 7 days post-surgery. Mice were monitored daily for signs of distress, wound healing, and overall health status.

1.3 HIIT regimen

The HIIT regimen was adapted from a previously published protocol (Henriquez-Olguin et al. 2019). Training sessions were conducted on a motorized treadmill during the dark period of the circadian cycle, beginning no earlier than 10:00 AM and ending no later than 5:00 PM. Each bout of training consisted of 60 seconds of running at 10° incline and 90%-110% of maximal running speed (MRS) followed by a 30-second passive recovery period, and each training session included 15 bouts of training bracketed by warm-up and cool-down periods, when the animals were run for 5 minutes at 10 m/minute. Training sessions were performed once per day on three non-consecutive days per week for six weeks, and running speed was increased by 1 m/minute each week to ensure that training intensity remained high throughout the 6-week period. If a mouse appeared to slow its running pace and/or began to drift toward the back of the treadmill belt, a mild electric shock was applied to restore the animal’s running speed and treadmill position. Mice in all groups were monitored daily for signs of fatigue, distress, or injury.

1.4 Familiarization with the treadmill apparatus

Treadmill speed was set at 5 m/minute with 0° incline, and the mice were run 5 minutes/day for 5 days; then, surgical procedures were performed in the MI-only, MI+HIIT, and Sham groups, and animals in all groups remained sedentary for one week before the HIIT regimen was initiated in the HIIT-only and MI+HIIT groups (Henriquez-Olguin et al. 2019, Knudsen et al. 2020).

1.5 Exercise capacity

Maximum running capacity was measured on an 8-lane DB030 treadmill (Zhishuduobao biological company, Beijing) via a protocol modified from previous publications (Marcinko et al. 2015, Knudsen et al. 2020). Animals ran at 10° incline and 10 m/minute for 10 minutes; then, the speed was increased by 2 m/minute every 3 minutes until exhaustion, which was defined as the inability to remain on the treadmill for >5 sec. Animals were removed immediately following exhaustion. The maximal speed reached was taken as the maximal running speed (MRS), and the corresponding total running distance at exhaustion was defined as the maximal running capacity (MRC); time to exhaustion was also recorded.

1.6 Echocardiography

Echocardiography was performed using a Vevo 2100 high-resolution imaging system (FUJIFILM VisualSonics, Toronto, Canada) equipped with an MS-550D 40-MHz linear-array transducer. Mice were lightly anesthetized with 1–1.5% inhaled isoflurane, and heart rates were maintained between

450–550 beats/min; LV images were acquired from the long-axis view at the papillary muscle level over three consecutive cardiac cycles.

1.7 5-ethynyl-2'-deoxyuridine (EdU) incorporation

Mice in all groups received intraperitoneal injections of 10 mg/kg EdU in sterile saline at Week 3, Week 4, and Week 5 after the HIIT regimen was initiated in the MI+HIIT and HIIT-only groups (Liu et al. 2015, Patterson et al. 2017).

1.8 Sample acquisition for immunofluorescence and RNA sequencing

Mice were euthanized under deep isoflurane anesthesia followed by decapitation to ensure rapid and humane euthanasia. All procedures were approved by the Committee for the Ethics of Animal Experiments at Henan University (HUSOM2022-153). At the Week-6 harvest, hearts were prospectively allocated to immunofluorescence (histology) or RNA extraction to avoid cross-use of tissue. For immunofluorescence, whole hearts were excised, rinsed in ice-cold PBS, and fixed overnight in 4% paraformaldehyde; left-ventricular (LV) tissue was then processed for paraffin embedding and staining as described. For RNA sequencing, hearts were collected from a separate, pre-assigned subset; after excision and a brief ice-cold PBS rinse, the atria, right ventricle, and great vessels were removed, and the whole LV was processed unfixed and rapidly snap-frozen in liquid nitrogen and stored at −80 °C until RNA isolation. Group allocation was balanced across experimental arms.

1.9 Immunofluorescent staining

After overnight fixation in 4% paraformaldehyde (see Tissue Collection and Allocation), hearts were processed and embedded in paraffin. Serial sections (5-μm thickness) were cut from the paraffin blocks and mounted on positively charged slides. EdU incorporation was detected with a Click-iT® Plus EdU Imaging Kit (Thermo Fisher Scientific, Cat. No. C10640) as directed by the manufacturer's instructions with modifications: sections were deparaffinized in xylene (twice for 10 minutes) and rehydrated with progressively lower concentrations of ethanol (100%, 95%, 70%, 50%, and distilled water, 5 minutes each). Slides were incubated in citrate-based antigen retrieval solution (Thermo Fisher Scientific, Cat. No.005000) at 95°C for 20 minutes and cooled to room temperature; then, the sections were permeabilized with 0.5% Triton X-100 (Thermo Fisher Scientific, Cat. No.85111) in PBS for 20 minutes and blocked with BlockAid solution (Thermo Fisher Scientific, Cat. No. B10710) for 30 minutes at room temperature. The Click-iT® reaction cocktail was prepared as stated in the manufacturer's instructions and applied to the sections at room temperature for 30 minutes while protected from light; then, the sections were washed 3 times with PBS. To visualize cardiomyocytes, sections were incubated overnight at 4 °C with primary antibodies against cardiac troponin T (cTnT, Thermo Fisher Scientific, Cat. No. MA5-35608, 1:200 dilution) diluted in BlockAid solution, washed with PBS, and incubated with a fluorophore-conjugated secondary antibody (Alexa Fluor® 488, Thermo Fisher Scientific, Cat. No. A-11008, 1:500 dilution) diluted in PBS for 1 hour at room temperature in the dark. Nuclei were counterstained with Hoechst 33342 (Thermo Fisher Scientific, Cat. No. C10640, 1:2000 dilution) diluted in PBS for 5 minutes, and sections were mounted with anti-fade mounting medium (Thermo Fisher Scientific, Cat. No. P36965).

1.10 Masson's Trichrome staining

Paraffin-embedded heart tissues were cut to 5-μm–thick sections and stained with a Masson's Trichrome staining Kit (Solarbio, G1346) as directed by the manufacturer's instructions. Images were

obtained with a biological microscope (Leica DM3000, Germany), and the ratio of fibrotic area to total area was determined with ImageJ software.

1.11 Bulk RNA-sequencing analysis

Total RNA was extracted from fresh-frozen whole LV tissue using an RNeasy Mini Kit (Qiagen) with on-column DNase digestion. RNA integrity was confirmed (RIN≥8), and libraries were prepared with a TruSeq Stranded mRNA Library Prep Kit (Illumina) followed by paired-end sequencing (2×150 bp) on an Illumina NovaSeq 6000 platform; 20–30 million reads were obtained per sample. Raw reads were quality-checked via FastQC, trimmed with Trimmomatic, and then aligned to the mouse reference genome (GRCm39) via STAR. Gene-level quantification was performed with featureCounts, and differential expression analysis was conducted via DESeq2. Genes with adjusted $p<0.05$ and log2 fold change ≥1 were considered significant. Enrichment analysis for Gene Ontology (GO) terms and KEGG pathways was performed with clusterProfiler, and the results were visualized via ggplot2 and heatmap. The RNA-seq data generated in this study have been deposited in the NCBI Sequence Read Archive (SRA) under BioProject accession number PRJNA1390587.

1.12 Statistical analysis

Statistical analyses were performed with MATLAB software (version 2024a, MathWorks, Natick, MA), and results are presented as mean ± standard deviation (SD) unless otherwise specified. Normality and homogeneity of variance were evaluated with the Shapiro–Wilk test and Levene’s test, respectively. For single time-point comparisons across groups (e.g., Week 6 histological and molecular endpoints), group differences were assessed using one-way ANOVA. When a significant main effect of group was detected, post hoc pairwise comparisons between groups were performed using independent two-tailed t-tests, and the resulting p-values were adjusted for multiple testing using the Benjamini–Hochberg false discovery rate (FDR) procedure. For variables measured at two time points in the same animals (exercise capacity and echocardiographic parameters at Week 0 and Week 6), two-way repeated-measures ANOVA was additionally performed with group (Control, HIIT-only, Sham, MI-only, and MI+HIIT) as the between-subject factor, time (Week 0 vs. Week 6) as the within-subject factor, and a group × time interaction term. When indicated, post hoc pairwise comparisons at specific time points were conducted with FDR-adjusted p-values as described above. A p-value or FDR-adjusted $p\text{-value} < 0.05$ was considered statistically significant.

2 Supplementary Figures and Tables

2.1 Supplementary Figures

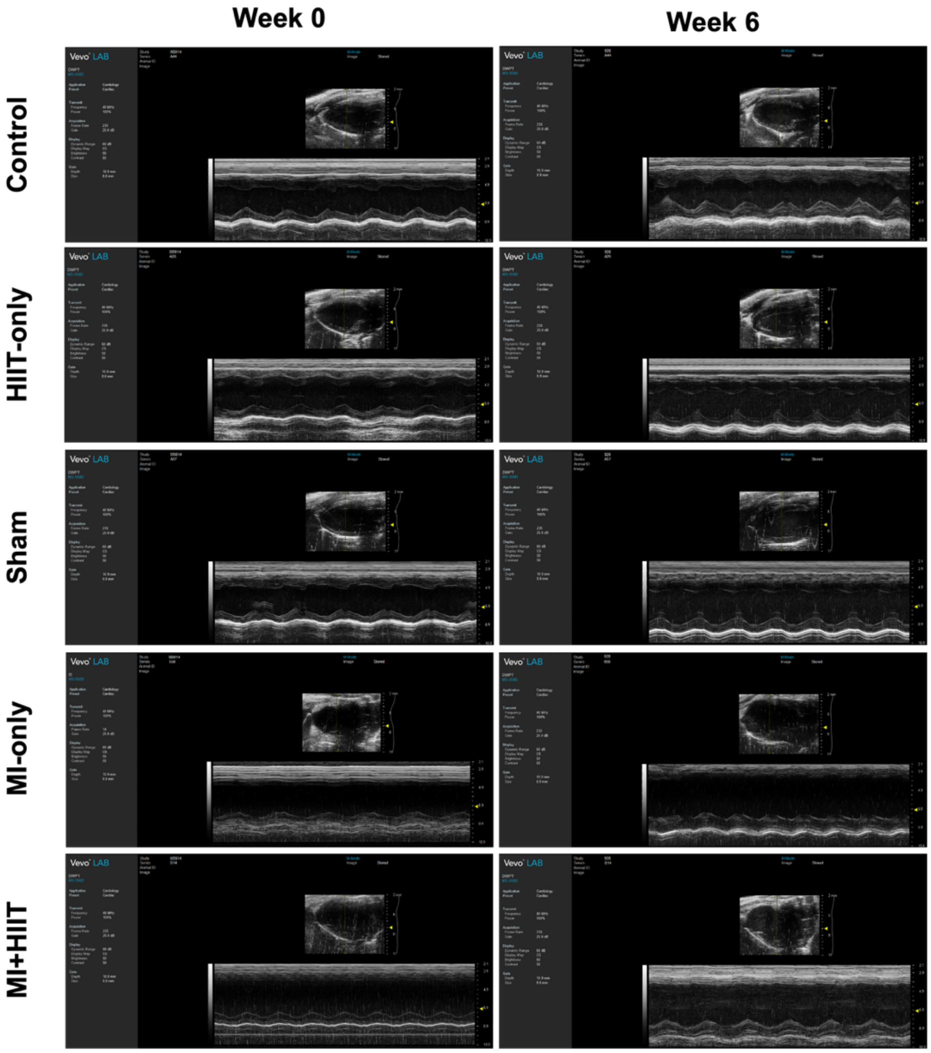

Supplemental Figure 1. Echocardiographic images were collected from each experimental group at Week 0 and Week 6. Con: Control, HIIT: high-intensity interval training, MI: myocardial infarction.

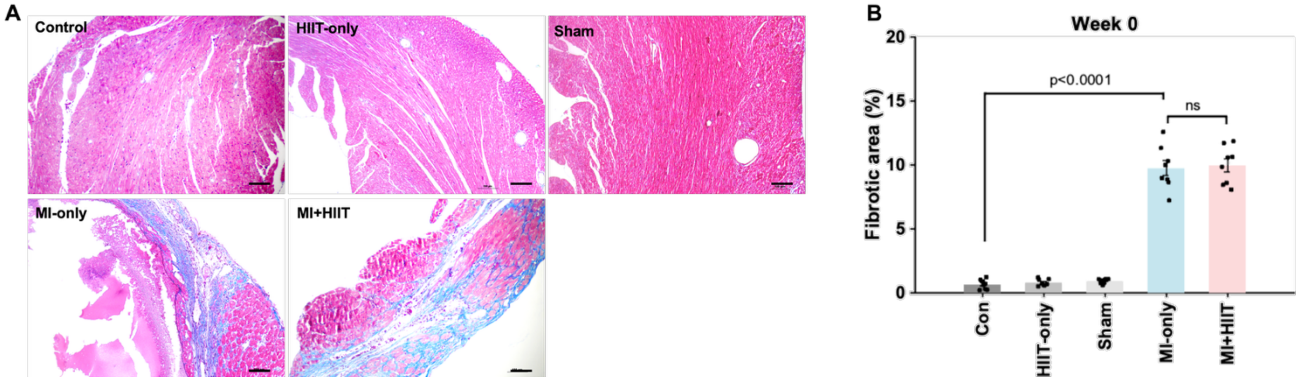

Supplemental Figure 2. Masson's trichrome staining of LV sections at Week 0 of each experimental group. Con: Control, HIIT: high-intensity interval training, MI: myocardial infarction. (A) Cardiac tissue sections were obtained from the LVs of mice sacrificed at Week 0 and stained with Masson's trichrome to identify fibrotic (blue) and nonfibrotic (red) regions; then, (B) the ratio of the area of fibrosis to the area of the entire LV was calculated and presented as a percentage. Representative images are displayed in panel A (Scale bar=100 μm). Data are presented as mean ± SD. Group differences were evaluated using one-way ANOVA for comparisons across all five

experimental groups, followed by post hoc analysis where applicable. For comparisons between two groups, independent two-tailed t-tests were performed, with p-values adjusted for multiple comparisons using the Benjamini-Hochberg false discovery rate (FDR) correction. An adjusted p-value of less than 0.05 was considered statistically significant. (n=8 mice per experimental group).

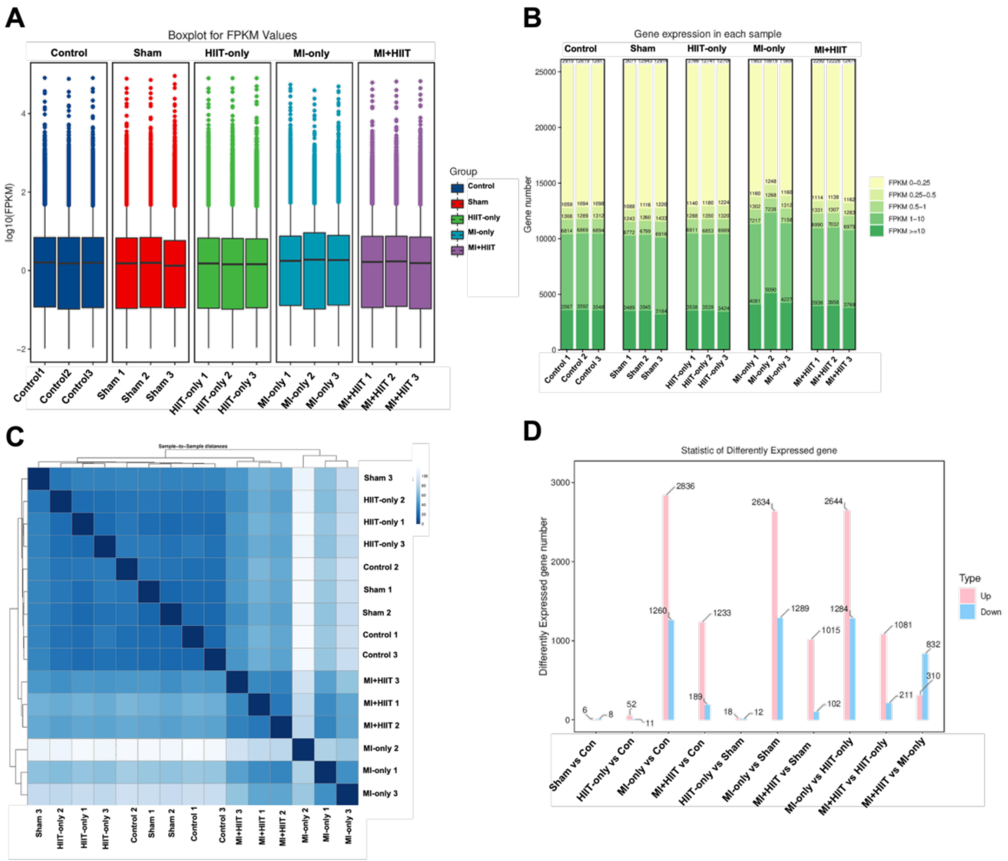

Supplemental Figure 3. Overview of RNA sequencing data analysis of each experimental group. (A) Boxplot of FPKM values for all genes in each group. Log10-transformed FPKM values are shown to illustrate the distribution of gene expression levels across the samples. Each group includes three biological replicates. (B) Stacked bar chart showing the distribution of gene expression levels in each sample. Genes are categorized into four expression ranges based on their FPKM values: 0-0.25, 0.25-0.5, 0.5-1, and ⩾1. This visualization highlights the proportion of highly expressed genes (FPKM ⩾1) in each group. (C) Heatmap of sample-to-sample correlation based on transcriptomic profile. Hierarchical clustering demonstrates the similarity of gene expression patterns within and between groups. (D) Bar chart of the number of differentially expressed genes (DEGs) between pairs of groups. The DEGs are categorized as upregulated (pink) or downregulated (blue) based on log2 fold-change and statistical significance ($P<0.05$). RNA-seq sample were derived from fresh-frozen whole LV tissue. *Con: Control.*

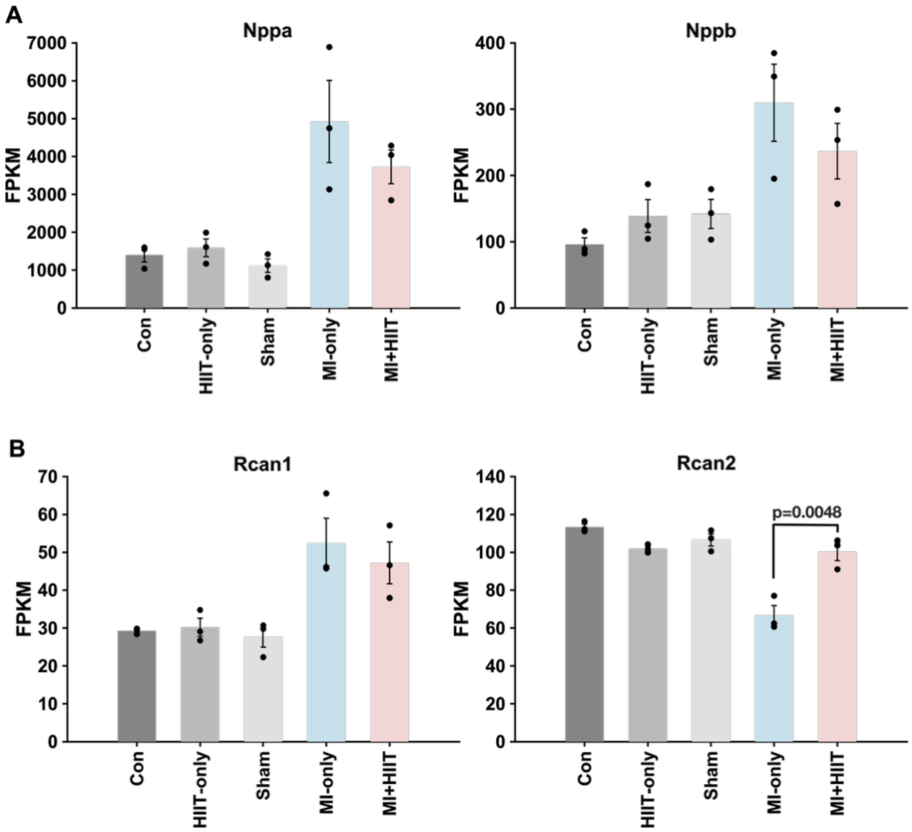

Supplemental Figure 4. FPKM normalization of bulk RNA sequencing data of cardiac hypertrophy related gene expression in each experimental group. Bulk RNA sequencing data were normalized according to Fragments Per Kilobase of transcript per Million mapped reads (FPKM), and results are displayed for (A) marker of cardiac hypertrophy (Nppa, Nppb), (B) inhibitor of cardiac hypertrophy (Rcan1, Rcan2). Data are presented as mean ± SD; p-values were determined via independent two-tailed t-tests, with p-values adjusted for multiple comparisons using the Benjamini-Hochberg false discovery rate (FDR) correction. An adjusted p-value of less than 0.05 was considered statistically significant. (n=3 mice per experimental group).

2.2 Supplementary Tables

Supplemental Table 1. Maximum running capacity of each experimental group at Week 0

	Control (n=10)	HIIT-only (n=10)	Sham (n=8)	MI-only (n=7)	MI+HIIT (n=7)	P_{ANOVA}
**Distance (m)**	1180.20±63.5	1257.20±233.15	1222.30±200.96	887.63±213.69$^{\#}$	953.00±174.76$^{\#}$	0.0006
**Speed (m/min)**	32.00±1.15	32.30±2.54	31.88±2.64	26.83±3.19$^{\#}$	28.00±2.38$^{\#}$	5.80×10^{-5}
**Time (min)**	59.7±1.34	62.50±6.64	61.63±7.13	47.33±6.38$^{\#}$	52.86±6.36$^{\#}$	4.16×10^{-5}

Data are presented as mean±SD. $^{\#}$ p<0.05, $^{\#\#}$ p<0.01 compared to the Control group; *p<0.05, **p<0.01 compared to the MI-only group (independent two-sample t-test)

Supplemental Table 2. Maximum running capacity of each experimental group at Week 6

	Control (n=10)	HIIT-only (n=10)	Sham (n=8)	MI-only (n=7)	MI+HIIT (n=7)	P_{ANOVA}
**Distance (m)**	1123±201.2	1526±244.24$^{##}$	1184±195.28	568.00±345.31$^{#}$	1248.40±186.28*	1.29×10^{-7}
**Speed (m/min)**	30.70±2.54	35.80±2.90$^{##}$	32.00±2.45	21.17±6.77$^{#}$	32.57±2.76*	7.90×10^{-8}
**Time (min)**	59.2±6.23	71.3±6.40$^{##}$	60.88±6.47	35.33±17.56$^{#}$	62.57±5.80*	1.07×10^{-7}

Data are presented as mean±SD. $^{#}$ p<0.05, $^{##}$ p<0.01 compared to the Control group; *p<0.05, **p<0.01 compared to the MI-only group (independent two-sample t-test)

Supplemental Table 3. Echocardiography parameters of each experimental group at Week 0

	Control (n=9)	HIIT-only (n=9)	Sham (n=9)	MI-only (n=9)	MI+HIIT (n=9)	P_{ANOVA}
**LVEF (%)**	52.21±5.96	51.02±6.67	52.76±5.72	24.52±8.46$^{##}$	23.26±8.23$^{##}$	7.02×10^{-14}
**LVFS (%)**	26.21±3.67	25.55±3.95	26.54±3.95	11.28±4.04$^{##}$	10.59±3.93$^{##}$	7.65×10^{-14}
**IVSTd (mm)**	0.91±0.03	0.87±0.08	0.90±0.09	0.75±0.15	0.80±0.15	0.0122
**IVSTs (mm)**	1.17±0.07	1.11±0.09	1.15±0.06	0.97±0.22	0.96±0.24	0.0133
**LVIDd (mm)**	3.54±0.25	3.65±0.24	3.52±0.17	4.50±0.77$^{#}$	4.14±0.60$^{#}$	0.0001
**LVIDs (mm)**	2.61±0.24	2.73±0.31	2.58±0.15	4.02±0.83$^{##}$	3.71±0.68$^{##}$	7.81×10^{-8}
**LVPWd (mm)**	0.86±0.04	0.85±0.04	0.90±0.08	0.72±0.20	1.02±0.76	0.5079
**LVPWs (mm)**	1.05±0.06	1.07±0.10	1.10±0.08	0.80±0.25	0.86±0.19	0.0002
**LVVd (uL)**	52.66±9.23	56.76±8.76	51.75±6.32	77.98±28.59	95.85±35.91	0.0002
**LVVs (uL)**	25.23±5.41	28.23±7.67	24.35±3.41	74.54±33.10	61.41±29.03	1.38×10^{-6}

Data are presented as mean±SD. $^{#}$ p<0.05, $^{##}$ p<0.01 compared to the Control group; *p<0.05, **p<0.01 compared to the MI-only group (independent two-sample t-test). *EF: ejection fraction, FS: fractional shortening, LV: left ventricle, IVSTd: end-diastolic interventricular septum, IVSTs: end-systolic interventricular septum thickness, LVIDd: end-diastolic LV interventricular septum, LVIDs: end-systolic LV interventricular septum thickness, LVPWd: end-diastolic LV posterior wall thickness, LVPWs: end-systolic LV posterior wall thickness, LVVd: end-diastolic LV volume, LVVs: end-systolic LV volume.*

Supplemental Table 4. Echocardiography parameters of each experimental group at Week 6

	Control (n=9)	HIIT-only (n=9)	Sham (n=9)	MI-only (n=9)	MI+HIIT (n=9)	P_{ANOVA}
**LVEF (%)**	53.13±6.69	55.62±8.23	54.54±4.02	16.19±6.00##	26.18±8.63##*	5.98×10^{-17}
**LVFS (%)**	26.94±4.22	28.61±5.67	27.72±2.68	7.41±2.68##	12.24±4.26##*	1.43×10^{-15}
**IVSTd (mm)**	0.91±0.10	0.95±0.05	0.94±0.10	0.68±0.19	0.86±0.15	0.0003
**IVSTs (mm)**	1.25±0.08	1.27±0.10	1.25±0.11	1.08±0.30	0.81±0.25	1.73×10^{-5}
**LVIDd (mm)**	3.73±0.18	3.65±0.35	3.68±0.19	5.79±0.99##*	4.89±0.51##*	2.23×10^{-11}
**LVIDs (mm)**	2.27±0.23	2.62±0.39	2.66±0.17	5.38±1.04##*	4.30±0.63##*	7.65×10^{-14}
**LVPWd (mm)**	0.92±0.06	0.95±0.08	0.94±0.08	0.76±0.25	0.87±0.15	0.0596
**LVPWs (mm)**	1.15±0.13	1.22±0.09	1.21±0.11	0.86±0.25	0.96±0.21	9.49×10^{-5}
**LVVd (uL)**	59.51±6.73	57.00±13.56	57.50±6.97	172.08±73.64	113.67±28.81	1.77×10^{-8}
**LVVs (uL)**	28.03±5.77	25.81±8.83	26.15±4.04	146.61±73.20	85.53±31.52	4.39×10^{-9}

Data are presented as mean±SD. # p<0.05, ## p<0.01 compared to the Control group; *p<0.05, **p<0.01 compared to the MI-only group (independent two-sample t-test). *EF: ejection fraction, FS: fractional shortening, LV: left ventricle, IVSTd: end-diastolic interventricular septum, IVSTs: end-systolic interventricular septum thickness, LVIDd: end-diastolic LV interventricular septum, LVIDs: end-systolic LV interventricular septum thickness, LVPWd: end-diastolic LV posterior wall thickness, LVPWs: end-systolic LV posterior wall thickness, LVVd: end-diastolic LV volume, LVVs: end-systolic LV volume.*

Supplemental Table 5. Heart weights and bodyweights of each experimental group

	Control (n=9)	HIIT-only (n=10)	Sham (n=10)	MI-only (n=8)	MI+HIIT (n=10)	P_{ANOVA}
**Body weight Week 0 (g)**	20.79±0.86	20.68±0.91	20.76±0.47	19.84±0.53#	19.85±0.76	0.0059
**Body weight Week 6 (g)**	27.93±1.11	27.43±1.40	29.01±1.34	29.16±0.52##	26.83±1.79*	0.0014
**Heart weight Week 6 (g)**	0.17±0.05	0.22±0.06	0.18±0.03	0.25±0.05#	0.30±0.04##*	7.82×10^{-7}
**HW/BW, Week 6 (%)**	0.61±0.19	0.80±0.21	0.62±0.13	0.85±0.15#	1.13±0.15##**	8.63×10^{-8}

Data are presented as mean ± SD. # p<0.05, ## p<0.01 compared to the Control group; *p<0.05, **p<0.01 compared to the MI-only group (independent two-sample t-test). *HW/BW: heart-weight:body weight ratio.*

Supplemental Table 6. Infarct size (fibrotic area %) at Week 0 and Week 6 of each experimental group

**Fibrotic area (%)**	Control (n=8)	HIIT-only (n=8)	Sham (n=8)	MI-only (n=8)	MI+HIIT (n=8)	P_{ANOVA}
**Week 0**	0.66±0.38	0.80±0.26	0.93±0.19	9.74±1.68##	9.97±1.47##	p<0.001
**Week 6**	0.65±0.39	1.32±0.51	0.94±0.27	13.16±2.18##	8.84±1.36##**	p<0.001

Supplemental Table 7. Quality control and preprocessing of RNA sequencing data of each experimental group

Sample	RawReads(M)	RawBases(G)	CleanReads(M)	CleanBases(G)	ValidBases(%)	Q30(%)	GC(%)
**Control 1**	51.71	7.38	48.89	6.97	94.54	97.68	45.88
**Control 2**	50.92	7.28	48.28	6.9	94.81	97.53	46.61
**Control 3**	51.55	7.32	48.5	6.89	94.09	97.58	45.76
**HIIT-only 1**	50.81	7.27	48.24	6.9	94.94	97.36	45.96
**HIIT-only 2**	52.06	7.44	49.37	7.06	94.83	97.56	45.84
**HIIT-only 3**	52.97	7.52	49.83	7.08	94.06	97.54	45.3
**Sham 1**	51.73	7.35	48.65	6.91	94.03	97.64	46.01
**Sham 2**	52.65	7.42	49.15	6.92	93.35	97.76	46.04
**Sham 3**	50.76	7.29	48.42	6.95	95.39	97.47	44.95
**MI-only 1**	51.17	7.3	48.37	6.9	94.53	97.47	47.3
**MI-only 2**	51.61	7.35	48.69	6.93	94.35	97.53	48.03
**MI-only 3**	51.2	7.3	48.3	6.89	94.35	97.61	47.87
**MI+HIIT 1**	52.61	7.42	49.07	6.92	93.28	97.57	46.78
**MI+HIIT 2**	51.86	7.38	49.01	6.98	94.49	97.62	46.5
**MI+HIIT 3**	51.73	7.39	48.92	6.98	94.56	97.32	46.35

Q30(%): percentage of bases with Phred score ⩾30, GC(%): GC content.

Supplemental Table 8. Alignment rate statistics of reads to the reference genome mapping of each experimental group

Sample	Total reads	Total mapped reads	Multiple mapped	Uniquely mapped	Read-1	Read-2	Reads map to '+'	Reads map to '-'	Non-splice reads	Splice reads	Reads mapped in proper pairs
**Control 1**	48886552	48357483(98.92%)	10867654(22.23%)	37489829(76.69%)	18785239(38.43%)	18704590(38.26%)	18730552(38.31%)	18759277(38.37%)	22842774(46.73%)	14647055(29.96%)	37176780(76.05%)
**Control 2**	48279622	47741602(98.89%)	9573431(19.83%)	38168171(79.06%)	19130571(39.62%)	19037600(39.43%)	19070028(39.50%)	19098143(39.56%)	23016776(47.67%)	15151395(31.38%)	37836478(78.37%)
**Control 3**	48502154	47906358(98.77%)	10482974(21.61%)	37423384(77.16%)	18756862(38.67%)	18666522(38.49%)	18695021(38.54%)	18728363(38.61%)	23362652(48.17%)	14060732(28.99%)	37074262(76.44%)
**HIIT-only 1**	48242058	47679417(98.83%)	10594931(21.96%)	37084486(76.87%)	18593458(38.54%)	18491028(38.33%)	18521953(38.39%)	18562533(38.48%)	22754288(47.17%)	14330198(29.70%)	36739134(76.16%)
**HIIT-only 2**	49371098	48834002(98.91%)	11842812(23.99%)	36991190(74.92%)	18538200(37.55%)	18452990(37.38%)	18482442(37.44%)	18508748(37.49%)	22566774(45.71%)	14424416(29.22%)	36682402(74.30%)
**HIIT-only 3**	49826248	49216488(98.78%)	11559626(23.20%)	37656862(75.58%)	18872199(37.88%)	18784663(37.70%)	18810372(37.75%)	18846490(37.82%)	23793794(47.75%)	13863068(27.82%)	37299420(74.86%)
**Sham 1**	48647052	48140474(98.96%)	10435392(21.45%)	37705082(77.51%)	18895249(38.84%)	18809833(38.67%)	18838083(38.72%)	18866999(38.78%)	23128904(47.54%)	14576178(29.96%)	37399358(76.88%)
**Sham 2**	49147028	48594106(98.87%)	10403405(21.17%)	38190701(77.71%)	19129328(38.92%)	19061373(38.78%)	19084685(38.83%)	19106016(38.88%)	23446769(47.71%)	14743932(30.00%)	37911112(77.14%)
**Sham 3**	48417280	47921268(98.98%)	14149734(29.22%)	33771534(69.75%)	16926565(34.96%)	16844969(34.79%)	16869207(34.84%)	16902327(34.91%)	21038162(43.45%)	12733372(26.30%)	33455846(69.10%)
**MI-only 1**	48371844	47783478(98.78%)	12395446(25.63%)	35388032(73.16%)	17737160(36.67%)	17650872(36.49%)	17682081(36.55%)	17705951(36.60%)	21384906(44.21%)	14003126(28.95%)	35092724(72.55%)
**MI-only 2**	48693760	48059197(98.70%)	7347034(15.09%)	40712163(83.61%)	20409240(41.91%)	20302923(41.70%)	20345607(41.78%)	20366556(41.83%)	24092634(49.48%)	16619529(34.13%)	40376114(82.92%)
**MI-only 3**	48303582	47799579(98.96%)	11883863(24.60%)	35915716(74.35%)	17997527(37.26%)	17918189(37.09%)	17948070(37.16%)	17967646(37.20%)	21447082(44.40%)	14468634(29.95%)	35651356(73.81%)
**MI+HIIT 1**	49074832	48510499(98.85%)	10458760(21.31%)	38051739(77.54%)	19067041(38.85%)	18984698(38.69%)	19014273(38.75%)	19037466(38.79%)	23120282(47.11%)	14931457(30.43%)	37741468(76.91%)
**MI+HIIT 2**	49007954	48464378(98.89%)	10350229(21.12%)	38114149(77.77%)	19105954(38.99%)	19008195(38.79%)	19043457(38.86%)	19070692(38.91%)	23134593(47.21%)	14979556(30.57%)	37792340(77.11%)
**MI+HIIT 3**	48917710	48315609(98.77%)	10447143(21.36%)	37868466(77.41%)	18988113(38.82%)	18880353(38.60%)	18914244(38.67%)	18954222(38.75%)	22997317(47.01%)	14871149(30.40%)	37504562(76.67%)

References

Henriquez-Olguin, C., L. B. Renani, L. Arab-Ceschia, S. H. Raun, A. Bhatia, Z. Li,T. E. Jensen (2019). Adaptations to high-intensity interval training in skeletal muscle require NADPH oxidase 2. *Redox Biol* **24**, 101188. DOI: 10.1016/j.redox.2019.101188

Knudsen, N. H., K. J. Stanya, A. L. Hyde, M. M. Chalom, R. K. Alexander, Y. H. Liou,C. H. Lee (2020). Interleukin-13 drives metabolic conditioning of muscle to endurance exercise. *Science* **368**(6490). DOI: 10.1126/science.aat3987

Liu, X., J. Xiao, H. Zhu, X. Wei, C. Platt, F. Damilano,A. Rosenzweig (2015). miR-222 is necessary for exercise-induced cardiac growth and protects against pathological cardiac remodeling. *Cell Metab* **21**(4), 584–595. DOI: 10.1016/j.cmet.2015.02.014

Marcinko, K., S. R. Sikkema, M. C. Samaan, B. E. Kemp, M. D. Fullerton and G. R. Steinberg (2015). High intensity interval training improves liver and adipose tissue insulin sensitivity. *Mol Metab* **4**(12), 903–915. DOI: 10.1016/j.molmet.2015.09.006

Patterson, M., L. Barske, B. Van Handel, C. D. Rau, P. Gan, A. Sharma,H. M. Sucov (2017). Frequency of mononuclear diploid cardiomyocytes underlies natural variation in heart regeneration. *Nat Genet* **49**(9), 1346–1353. DOI: 10.1038/ng.3929
